# Plumbagin inhibits invasion and migration of breast and gastric cancer cells by downregulating the expression of chemokine receptor CXCR4

**DOI:** 10.1186/1476-4598-10-107

**Published:** 2011-09-01

**Authors:** Kanjoormana Aryan Manu, Muthu K Shanmugam, Peramaiyan Rajendran, Feng Li, Lalitha Ramachandran, Hui Sin Hay, Radhamani Kannaiyan, Shivananju Nanjunda Swamy, Shireen Vali, Shweta Kapoor, Bhargavi Ramesh, Pradeep Bist, Evelyn S Koay, Lina HK Lim, Kwang Seok Ahn, Alan Prem Kumar, Gautam Sethi

**Affiliations:** 1 Department of Pharmacology, Yong Loo Lin School of Medicine, National University of Singapore, Singapore 117597; 2 Cancer Science Institute of Singapore, National University of Singapore, 28 Medical Drive, Singapore 117456; 3 Cellworks Group Inc., Saratoga, California, 95070; USA; Cellworks Research India Pvt. Ltd, Bangalore, 560066, India; 4 Department of Physiology, Yong Loo Lin School of Medicine, National University of Singapore, Singapore 117597; 5 Immunology Program, National University of Singapore, Singapore 117597; 6 Department of Pathology, National University of Singapore, Singapore 117597; 7 College of Oriental Medicine, Kyung Hee University, Seoul 130-701, Republic of Korea; 8 School of Anatomy and Human Biology, The University of Western Australia, Crawley, Perth, Western Australia 6009

## Abstract

**Background:**

Increasing evidence indicates that the interaction between the CXC chemokine receptor-4 (CXCR4) and its ligand CXCL12 is critical in the process of metastasis that accounts for more than 90% of cancer-related deaths. Thus, novel agents that can downregulate the CXCR4/CXCL12 axis have therapeutic potential in inhibiting cancer metastasis.

**Methods:**

In this report, we investigated the potential of an agent, plumbagin (5-hydroxy-2-methyl-1, 4-naphthoquinone), for its ability to modulate CXCR4 expression and function in various tumor cells using Western blot analysis, DNA binding assay, transient transfection, real time PCR analysis, chromatin immunoprecipitation, and cellular migration and invasion assays.

**Results:**

We found that plumbagin downregulated the expression of CXCR4 in breast cancer cells irrespective of their HER2 status. The decrease in CXCR4 expression induced by plumbagin was not cell type-specific as the inhibition also occurred in gastric, lung, renal, oral, and hepatocellular tumor cell lines. Neither proteasome inhibition nor lysosomal stabilization had any effect on plumbagin-induced decrease in CXCR4 expression. Detailed study of the underlying molecular mechanism(s) revealed that the regulation of the downregulation of CXCR4 was at the transcriptional level, as indicated by downregulation of mRNA expression, inhibition of NF-κB activation, and suppression of chromatin immunoprecipitation activity. In addition, using a virtual, predictive, functional proteomics-based tumor pathway platform, we tested the hypothesis that NF-κB inhibition by plumbagin causes the decrease in CXCR4 and other metastatic genes. Suppression of CXCR4 expression by plumbagin was found to correlate with the inhibition of CXCL12-induced migration and invasion of both breast and gastric cancer cells.

**Conclusions:**

Overall, our results indicate, for the first time, that plumbagin is a novel blocker of CXCR4 expression and thus has the potential to suppress metastasis of cancer.

## Background

Metastasis is a highly complex and non-spontaneous process that generally affects vital organs such as brain, lung, liver, bone or lymph nodes [1-3] in the later stages of cancer progression. At present, there are no approved drugs that can specifically target the metastatic process [4,5], and little is known about the molecular mechanism(s) regulating the process of metastasis [5]. Chemokines are a large family of small chemotactic cytokines that regulate multiple biological processes such as leukocyte trafficking, hematopoiesis, adhesion, and angiogenesis [6-8]. Based on the position of the first two conserved cysteine residues, the chemokines can be classified into four subfamilies, CXC, CC, C, and CX3C, and act on leukocytes via selective membrane-bound G protein-coupled receptors [2,3].

It has been well documented that the CXCL12/CXCR4 signaling cascade plays a crucial role in cancer proliferation, migration and metastasis [9]. CXCR4 is expressed by various types of tumor cells, including breast [10], colorectal [11], gastric [12], ovarian [13], pancreatic [14], prostate [15], lung [16], melanoma [17], and brain [18] tumor cells. The SDF-1α/CXCR4 attraction leads breast cancer cells to leave the circulation and migrate into organs that express large amounts of chemokines, where the cancer cells proliferate, induce angiogenesis and form metastatic tumors [7,19]. As CXCR4 expression has been correlated with poor overall survival rate in patients with breast cancer [20], and colorectal cancer [21], CXCR4 has been considered as a potential therapeutic target for inhibiting cancer metastasis [22].

In the present report, we studied the effect of plumbagin (5-hydroxy-2-methyl-1, 4-naphthoquinone, an analogue of vitamin K3) as a novel regulator of the CXCL12/CXCR4 signaling axis. Plumbagin, a naturally occurring yellow pigment predominantly found in the plants of the *Plumbaginaceae, Ancestrocladaceae*, and *Dioncophyllaceae *families, has been reported to exhibit significant anti-proliferative, pro-apoptotic, chemopreventing and radiosensitizing activities in different tumor cells and animal models [23-30]. Because CXCR4 is known to mediate proliferation, invasion and metastasis of tumor cells, we postulated that plumbagin may modulate the expression of CXCR4 and inhibit tumor cell invasion. Our results demonstrate, for the first time, that plumbagin can downregulate CXCR4 expression in various tumor cells, including HER2-overexpressing breast cancer cells, and this could be through its inhibition of NF-κB activation. We also found that plumbagin can significantly inhibit CXCL12-induced migration and invasion of breast and gastric tumor cells.

Alongside testing the effects of plumbagin experimentally in various tumor cells, we also tested the hypothesis of plumbagin-mediated NF-κB inhibition as the key reason for the reduction in CXCR4 and other metastatic genes, in a virtual, predictive, tumor cell system. The virtual epithelial tumor cell platform on which predictive NF-κB inhibition studies were conducted is a comprehensive integrated functional proteomics based, dynamic representation of the pathways representing the key cancer phenotypes of proliferation, apoptosis, angiogenesis, metastasis and conditions of tumor microenvironment including tumor-associated inflammation [31-33]. In this virtual tumor cell system, we can manipulate any protein or gene by over-expression or knockdown and get quantitative readouts and insights on the impact of this change on the various markers in the system. This predictive analysis helps in reconfirming the experimental hypothesis and giving mechanistic insights into understanding the trends in biomarker and phenotype changes. This novel approach has facilitated the analysis of the impact of plumbagin on metastatic genes based on the premise that this quinone mediates its affects primarily via modulation of NF-κB activation. The combination of predictive experiments coupled with guided experimental validations provide a more integrated analysis and a better understanding of the efficacy and mechanisms of action of specific anti-cancer drugs on physiological endpoints.

## Methods

### Reagents

Plumbagin, Tris, glycine, NaCl, SDS, lactacystin, and chloroquine were purchased from Sigma-Aldrich (St. Louis, MO, USA). Plumbagin was dissolved in dimethylsulfoxide as a 20 mM stock solution and stored at 4°C. Further dilution was done in cell culture medium. RPMI 1640, DMEM, fetal bovine serum (FBS), 0.4% trypan blue vital stain, antibiotic-antimycotic mixture, and HRP-conjugated secondary antibodies were obtained from Invitrogen (Carlsbad, CA, USA). Antibodies against CXCR4 and HER2 were obtained from Abcam (Cambridge, MA, USA). CXCL12 was purchased from ProSpec-Tany TechnoGene Ltd. (Rehovot, Israel).

### Cell Lines

Human breast cancer MDA-MB-231, BT474, and oral adenosquamous carcinoma CAL27 cells were obtained from American Type Culture Collection (Manassass, VA, USA).

AGS, MKN45, and SNU16 (gastric cancer) cells were kindly provided by Prof. Patrick Tan, DUKE-NUS Graduate Medical School, Singapore. Hep3B (hepatocellular carcinoma) cells were kindly provided by Prof. Hui Kam Man, National Cancer Centre, Singapore. 786-O (renal cell carcinoma) cells were kindly provided by Dr. John Yuen, Singapore General Hospital, Singapore. H1299 (lung adenocarcinoma) cells were kindly provided by Prof. Bharat B. Aggarwal, M.D. Anderson Cancer Center, Houston, TX, USA. MDA-MB-231, AGS, MKN45, SNU16, 786-O, and H1299 cells were cultured in RPMI 1640 medium with 10% FBS, 100 U/mL penicillin, and 100 μg/mL streptomycin. BT474 cells were cultured in DMEM F12 medium with 10% FBS, 100 U/mL penicillin, and 100 μg/mL streptomycin. Hep3B cells were cultured in DMEM supplemented with 10% FBS, 100 U/mL penicillin, and 100 μg/mL streptomycin. CAL27 cells were cultured in DMEM containing 10% FBS, and 1 mM pyruvate, 100 U/mL penicillin, and 100 μg/mL streptomycin and were maintained at 37°C in an atmosphere of 5% CO_2_-95% air.

### Western blot analysis

For detection of CXCR4 and HER2, plumbagin-treated whole-cell extracts were lysed in lysis buffer (20 mM Tris (pH 7.4), 250 mM NaCl, 2 mM EDTA (pH 8.0), 0.1% Triton X-100, 0.01 mg/mL aprotinin, 0.005 mg/mL leupeptin, 0.4 mM PMSF, and 4 mM NaVO_4_). Lysates were then spun at 14,000 rpm for 10 min to remove insoluble material and resolved on a 10% SDS gel. After electrophoresis, the proteins were electrotransferred to a nitrocellulose membrane, blocked with 5% nonfat milk to minimize non-specific binding, and probed with anti-CXCR4 or HER2 antibodies (1:3000) overnight at 4°C. The blot was washed, exposed to HRP-conjugated secondary antibodies for 2 h, and the CXCR4/HER2 expression was detected by chemiluminescence emission (ECL; GE Healthcare, Little Chalfont, Buckinghamshire, UK). The densitometric analysis of the scanned blots was done using Image J software and the results are expressed as fold change relative to the control.

### Nuclear extract preparation

Nuclear extracts were prepared at various time points after treatment for subsequent NF-κB DNA-binding activity assay. Cell nuclear fractions were extracted using a nuclear extraction kit (Active Motif, Carlsbad, CA, USA). Briefly, cells were washed, collected in ice-cold PBS in the presence of phosphatase inhibitors, and then centrifuged at 300 *g *for 5 min. Cell pellets were resuspended in a hypotonic buffer, treated with detergent, and centrifuged at 14,000 *g *for 30 s. After collection of the cytoplasmic fraction, the nuclei were lysed, and nuclear proteins were solubilized in lysis buffer and protein concentrations were determined by the Bradford protein assay (Bio-Rad Laboratories, Hercules, CA, USA).

### NF-κB DNA-binding activity assay

NF-κB DNA-binding activity was analyzed using the TransAM NF-κB p65 transcription factor assay kit (Active Motif, Carlsbad, CA, USA), following the manufacturer's instructions. Briefly, nuclear extracts (5 μg) from plumbagin-treated cells were incubated in a 96-well plate coated with oligonucleotide containing the NF-κB consensus-binding sequence 5'-GGGACTTTCC-3'. Bound NF-κB was then detected by a specific primary antibody. An HRP-conjugated secondary antibody was then applied to detect the bound primary antibody and provided the basis for colorimetric quantification. The enzymatic product was measured at 450 nm with a microplate reader (Tecan Systems, San Jose, CA, USA).

### NF-κB luciferase reporter assay

MDA-MB-231 cells were plated in 96-well plates with 1 × 10^4 ^cells per well in 10% FBS-supplemented RPMI medium. After overnight incubation, the cells were transfected with the NF-κB reporter plasmid linked to a luciferase gene or with the dominant-negative IκBα (IκBα-DN) plasmid. NF-κB luciferase plasmid was obtained from Stratagene (La Jolla, CA). Transfections were done according to the manufacturer's protocols using FuGENE-6 (Roche). At 24 h post-transfection, cells were treated with indicated concentrations of plumbagin for 2 h and then washed and lysed in luciferase lysis buffer (Promega), and the luciferase activity was measured with a luminometer using a luciferase assay kit (Promega) and was normalized to β-galactosidase activity. All the experiments were done in triplicates and repeated two or more times.

### RNA extraction and Real-time PCR analysis

Total RNA was extracted using the Trizol reagent (Invitrogen, Carlsbad, CA, USA), according to the manufacturer's instructions. Reverse transcription (RT) was then carried out at 37°C for 1 h. Each RT reaction contains 1 μg of total RNA, 1× RT buffer, 5 mM MgCl_2_, 425 μM each of dNTPs, 2 μM random hexamers, 0.35 U/μL RNase inhibitor, 1.1 U/μL MultiScribe reverse transcriptase and made up to 10 μL with sterile water. The relative expression of CXCR4 was then analyzed using quantitative RT-PCR (ABI PRISM 7500, Applied Biosystems, Foster City, CA, USA) with 18sRNA as an internal control. Primers and probes for human CXCR4 and 18sRNA were purchased as Assays-on-Demand kits (Applied Biosystems).

### Chromatin immunoprecipitation (ChIP) assay

The cells were processed for the ChIP assay as per the protocol described by Saccani et al., 2002 [34]. The antibody used for the ChIP was NF-kB (p65)Ab (Santa Cruz Biotechnology, SantaCruz, CA, USA). The sequence for human CXCR4 gene promoter was as follows: sense primer, 5'-ACAGAGAGACGCGTTCCTAG-3' and antisense primer, 5'-AGCCCAGGGGACCC TGCTG-3'. The PCR products were analyzed on 2% agarose gel electrophoresis and documented.

### Wound Healing Assay

MDA-MB-231 and AGS cells were treated with 5 μM plumbagin in RPMI medium containing 1% serum. Before plating the cells, two parallel lines were drawn at the underside of the wells, to serve as fiducial marks demarcating the wound areas to be analyzed. Prior to inflicting the wound, the cells should be fully confluent. The growth medium was aspirated off and replaced by calcium-free PBS to prevent killing of the cells at the edge of the wound by exposure to high calcium concentrations before two parallel scratch wounds were made perpendicular to the marker lines with a sterile 1000-μL automated pipette tip. Thereafter, the calcium-free PBS was then changed to medium with or without plumbagin. After incubation for 6 h, the growth medium was then changed to basal medium with or without CXCL12. 24 h later, the wounds were observed using bright field microscopy and multiple images were taken at areas flanking the intersections of the wound and the marker lines at the start and end of the experiment. Gap distance of the wound was measured at three different sites using Photoshop software, and the data were normalized to the average of the control. Graphs were plotted against the percentage of migration distance the cells moved before and after treatment, normalized to control.

### Invasion assay

The *in vitro *invasion assay was performed using Bio-Coat Matrigel invasion assay system (BD Biosciences, San Jose, CA, USA), according to the manufacturer's instructions. MDA-MB-231 and AGS cells (2 × 10^5 ^cells) were suspended in serum-free RPMI medium and seeded into the Matrigel transwell chambers consisting of polycarbonate membranes with 8-μm pores. After pre-incubation with or without plumbagin for 6 h, the transwell chambers were then placed into appropriate wells of a 24-well plate, in which either the basal medium only or basal medium containing CXCL12 had been added. After incubation for 24 h, the upper surfaces of the transwell chambers were wiped with cotton swabs and the invading cells were fixed and stained with crystal violet solution. The invading cell numbers were counted in five randomly selected microscope fields.

### Predictive Experiments on Virtual Tumor Cell

Predictive experiments were performed using the physiologically aligned and qualified Virtual Tumor Cell technology (Cellworks Group Inc (CWG), CA, USA [31,35]. The Cellworks Tumor cell platform provides a dynamic and transparent view of tumor cell physiology at the functional proteomics abstraction level. The platform's open-access architecture provides a framework for different 'what-if' analysis and studies in an automated high-throughput methodology.

### Platform description

**Figure 1:**
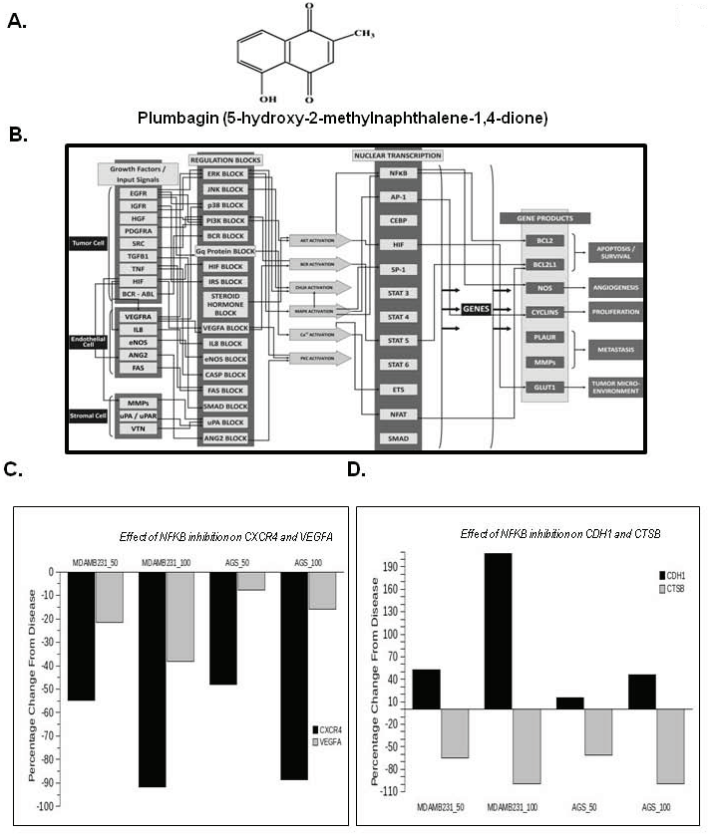
**Predictive *In Silico *Virtual tumor platform generated results**. **A**, The chemical structure of plumbagin. **B**, The figure illustrates a high-level view of the maze of interactions and cross-talks present in the Virtual Tumor Cell platform. ***C***, The figure illustrates the percentage reduction in CXCR4 and VEGFA with 50% and 100% inhibition of NF-κB in MDA-MB-231 (breast cancer) and AGS (gastric cancer) aligned virtual tumor cells. ***D***, The figure illustrates the percentage reduction in metastatic tumor markers- CDH1 and Cathepsin B with 50% and 100% inhibition of NF-κB activation in the two tumor baselines.

The virtual Tumor Cell Platform consists of a dynamic and kinetic representation of the signaling pathways underlying tumor physiology at the bio-molecular level. All the key relevant protein players and associated gene and mRNA species are comprehensively included in the system with their relationship quantitatively represented. Pathways and signaling for different cancer phenotypes comprise 75 major signaling networks with more than 3500 intracellular molecules and 12000 cross talks and links. The platform includes important signaling pathways comprising growth factors like EGFR, PDGFRA, FGFR, c-MET, VEGFR and IGF-1R, cell cycle regulators, mTOR signaling, p53 signaling cascade, cytokine pathways like IL1, IL4, IL6, IL12, TNF; TGF-b, hypoxia mediated regulation, angiogenic promoters, lipid mediators and tumor metabolism (Figure 1B). It has a wide coverage of kinases and transcription factors associated with tumor physiology network. The platform has been correlated against an extensive set of pre-defined *in vitro *and *in vivo *studies.

The starting control state of the system is based on normal epithelial cell physiology that is non-tumorigenic. The user can control the transition of the normal system to a neoplastic disease state aligning with specific tumor mutation profiles. This is accomplished as an example through over-expression of the tumorigenic genes like EGFR, IGF-1R; knock-downs of the tumor-suppressors like p53, PTEN; and increased states of hypoxia and oxidative stress. Knockdowns or over-expressions of biomolecular species can be done at the expression or activity levels. When a drug is introduced into the system with a specific mechanism of action, the drug concentration in the virtual experiments is explicitly assumed to be post ADME (Absorption, Distribution, Metabolism, and Excretion).

### Predictive Study Experimental Protocol

The virtual Tumor cell is simulated in the proprietary Cellworks computational backplane and initialized to a control state wherein all molecules attain the control steady state values, following which the triggers are introduced into the system.

The experiments were conducted in two different disease state baselines corresponding to MDA-MB-231 human breast cancer cell line and AGS human gastric cancer cell line. MDA-MB-231 baseline has an over-expression of BRAF, is KRAS dominant, P53 mutant and CDKN2A depleted cell line. AGS is a PI3KA over-expressed, KRAS dominant, β-catenin over-expressed and CDH1 (E-cadherin) depleted cell line. These baselines for MDA-MB-231 and AGS cell lines were created by overlaying the specific mutations on the control system to attain dynamic disease state. On these cell lines, NF-κB activation was inhibited by 50% and 100% to emulate plumbagin mediated NF-κB inhibition. The experiment is simulated and the system is allowed to dynamically settle to a different steady-state from the baseline and the biomarker trends evaluated as percentage change from baseline values with respect to 50% and 100% inhibition of NF-κB activation. The impact of NF-κB inhibition in these virtual cell lines on CXCR4, VEGFA and other metastatic markers was assayed and compared with experimental data for plumbagin.

### Statistical analysis

The experiments were carried out in triplicates and repeated at least twice. Data are expressed as the mean ± S.E.M. In all figures, vertical error bars denote the S.E.M. The significance of differences between groups was evaluated by Student's t-test and one way analysis of variance, (ANOVA). A p value of less than 0.05 was considered statistically significant.

## Results and Discussion

The present study was designed to investigate the effect of plumbagin (with structure shown in Figure 1A) on CXCR4 expression and also on cellular migration and invasion in various tumor cells.

### Predictive analysis of inhibition of NF-κB activation in tumor cells

To test whether inhibition of NF-κB activation is primarily causing the plumbagin-mediated impact on metastatic markers in tumor cells, we tested this hypothesis in the virtual tumor cells aligned to a breast cancer cell line MDA-MB-231 and a gastric cell line cell line AGS (Figure 1B). The inhibition of NF-κB activation by 50% and 100% leads to marked decrease in the expression of CXCR4 and slight decrease in VEGF expression in both these cell line profiles, as depicted in Figure 1C. These predictive results corroborate with the experimental data and support the hypothesis that plumbagin effects on metastatic phenotypes in tumor cells are mainly through inhibition of NF-κB activation. Additional metastatic markers such as Cathepsin B and E-Cadherin were also monitored. A predictive increase in CDH1 and a decrease in Cathepsin B (Figure 1D) also indicate that plumbagin would have a good impact on inhibiting metastasis phenotype in tumor cells.

### Plumbagin suppresses the expression of CXCR4 protein in breast cancer cells

**Figure 2:**
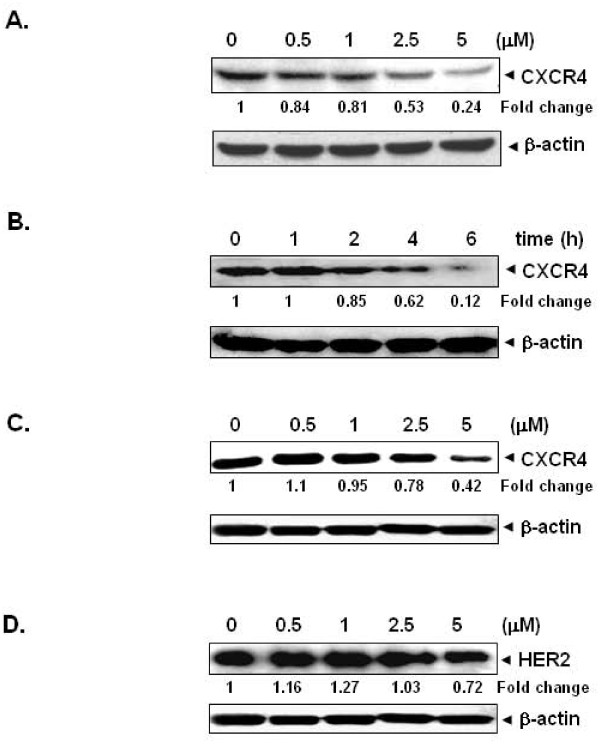
**Plumbagin suppresses CXCR4 expression in breast cancer cells**. ***A***, Plumbagin suppresses CXCR4 levels in a dose-dependent manner. MDA-MB-231 cells (1 × 10^6^) were treated with the indicated concentrations of plumbagin for 6 h. Whole-cell extracts were then prepared, and 30 μg of protein was resolved on SDS-PAGE, electrotransferred onto nitrocellulose membranes, and probed for CXCR4. The same blots were stripped and reprobed with β-actin antibody to show equal protein loading. ***B***, Plumbagin suppresses CXCR4 levels in a time-dependent manner. MDA-MB-231 cells (1 × 10^6^) were treated with 5 μM plumbagin for the indicated times, after which Western blotting was done as described above. The same blots were stripped and reprobed with β-actin antibody to show equal protein loading. ***C***, Plumbagin suppresses CXCR4 levels in HER2 overexpressing BT474 cells. BT474 cells (1 × 10^6^) were treated with the indicated concentrations of plumbagin for 6 h. Whole-cell extracts were then prepared, and 30 μg of protein was resolved on SDS-PAGE, electro-transferred onto nitrocellulose membranes, and probed for CXCR4. The same blots were stripped and reprobed with β-actin antibody to show equal protein loading. ***D***, Effect of plumbagin on HER2 expression in BT474 cells. BT474 cells (1 × 10^6^) were treated with the indicated concentrations of plumbagin for 6 h, after which Western blotting for HER2 was done as described above. The same blots were stripped and reprobed with β-actin antibody to show equal protein loading. The representative results of three independent experiments are shown.

Several lines of evidence implicate the role of CXCR4 in breast cancer metastasis [19,36]. Muller *et al*. found that motility and migration of breast cancer cells can be induced when they are exposed to their ligand, CXCL12 [36]. Also, breast cancer metastasis can be inhibited by silencing CXCR4 [37]. Hence, we first investigated the effect of plumbagin on CXCR4 expression in breast cancer MDA-MB-231 cells. When MDA-MB-231 cells were incubated with different concentrations of plumbagin for 6 h or with 5 μM of plumbagin for different times, plumbagin suppressed the expression of CXCR4 in a dose- and time-dependent manner (Figures 2A and 2B), confirming the predictive trends as in Figure 1C. HER2 overexpression has been linked with metastasis of breast cancer [38]. Furthermore, HER2 has been shown to induce the expression of CXCR4 in breast cancer cells [38]. Hence, we also investigated the effect of plumbagin in BT474 cells that express high endogenous levels of HER2 [39]. When BT474 cells were incubated with different concentrations of plumbagin for 6 h, the expression of CXCR4 was found to be down-regulated in a dose-dependent manner (Figure 2C). Since HER2 enhances the expression of CXCR4 by stimulating CXCR4 translation and attenuating CXCR4 degradation [40], we also examined whether plumbagin downregulates CXCR4 expression through regulation of HER2 expression. For this, HER2-overexpressing BT474 cells were incubated with different concentrations of plumbagin for 6 h and then examined for HER2 expression by Western blot analysis using specific antibodies. We found that HER2 expression was minimally affected after plumbagin treatment (Figure 2D), thus suggesting that downregulation of CXCR4 expression by plumbagin is not due to modulation of HER2 expression.

### Plumbagin downregulates CXCR4 in different tumor cell types

**Figure 3:**
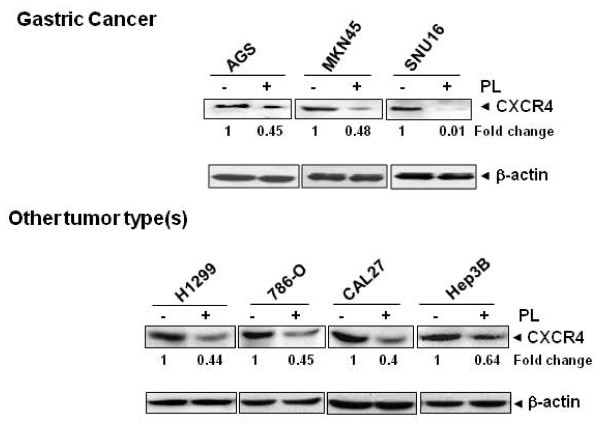
**Plumbagin downregulates CXCR4 in different tumor cell types. Gastric cancer (AGS, MKN45, and SNU16), lung adenocarcinoma (H1299), renal cell carcinoma (786-O), oral adenosquamous carcinoma (CAL27), hepatocellular carcinoma (Hep3B) cells were incubated with 5 μM plumbagin for 6 h**. Whole-cell extracts were prepared and analyzed by Western blot analysis using antibody against CXCR4. The same blots were stripped and reprobed with β-actin antibody to show equal protein loading. Representative results of three independent experiments are shown.

Up to this point, all of the afore-mentioned studies were carried out with breast cancer cell lines. However, CXCR4 is known to be overexpressed in a wide variety of tumor cells [41]. Hence, we carried out the same experiment to find out whether plumbagin downregulates expression of CXCR4 in gastric (AGS, MKN45, and SNU16) cancer cell lines, which has never been investigated before. Cells were treated with 5 μM plumbagin for 6 h before assessing the resultant effect on CXCR4 expression. Figure 3 clearly demonstrates that plumbagin substantially downregulated CXCR4 expression in all three gastric cancer cell lines, also confirming the predictive results from the virtual AGS cell line (Figure 1C). Upon further extension of studying the effect of plumbagin on CXCR4 expression in lung adenocarcinoma (H1299), renal cell carcinoma (786-O), oral adenosquamous carcinoma (CAL27), hepatocellular carcinoma (Hep3B) tumor cell lines, we also found that plumbagin dramatically downregulated the CXCR4 expression in all these cell lines (Figure 3). This showed convincingly that CXCR4 downregulation by plumbagin is not cell type-specific.

### Downregulation of CXCR4 expression by plumbagin is not mediated through its degradation

**Figure 4:**
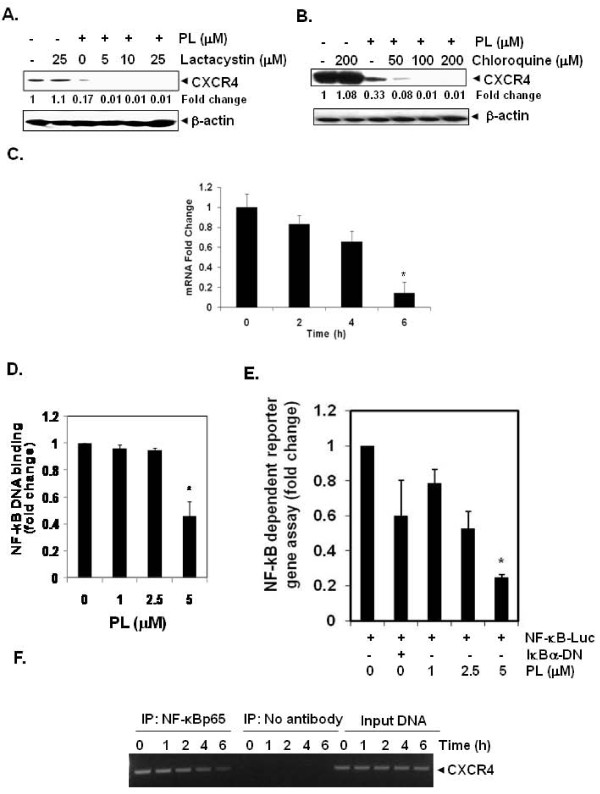
**Plumbagin suppresses CXCR4 through modulation of its mRNA level**. ***A and B***, Plumbagin suppresses CXCR4, through lysosomal but not proteosomal degradation. Cells were treated with indicated concentrations of lactacystin or chloroquine for 1 h at 37°C, followed by treatment of 5 μM plumbagin for 6 h. Whole-cell extracts were prepared and analyzed by Western blot analysis using antibodies against CXCR4. The same blots were stripped and reprobed with β-actin antibody to show equal protein loading. Representative results of three independent experiments are shown. ***C***, Plumbagin suppresses expression of CXCR4 mRNA in MDA-MB-231 cells. Cells were treated with 5 μM plumbagin for indicated times. Total RNA was isolated and analyzed by RT-PCR assay as described in Materials and Methods. 18S was shown to equal loading of total RNA. Representative results of three independent experiments are shown. ***D***, Plumbagin inhibits NF-κB activation in MDA-MB-231 breast cancer cells. MDA-MB-231 cells were incubated with indicated concentrations of plumbagin for 2 h. The nuclear extracts were assayed for NF-κB activation by TransAM p65 transcription factor assay kit. ***E***, MDA-MB-231 cells were transiently transfected with an NF-κB-luciferase plasmid and then treated with the indicated concentrations of plumbagin for 2 h. Cell supernatants were thereafter collected and assayed for luciferase activity as described in Materials and Methods. Representative results of three independent experiments are shown. Results are expressed as fold activity over the activity of the vector control. Bars indicate standard deviation. * indicates p value < 0.05). ***F***, Plumbagin inhibits binding of NF-κB to the CXCR4 promoter. MDA-MB-231 cells were treated with 5 μM plumbagin for indicated time intervals and the proteins were cross-linked with DNA by formaldehyde and then subjected to ChIP assay using an anti-p65 antibody with the CXCR4 primer. Reaction products were resolved by electrophoresis.

Because plumbagin could downregulate CXCR4 expression by enhancing its degradation, and CXCR4 has been shown to undergo ubiquitination at its lysine residue followed by degradation [42,43], we next investigated the possibility that plumbagin may enhance the rate of CXCR4 degradation via the activation of proteasomes. To determine this, we examined the ability of lactacystin, a proteasome inhibitor, to block plumbagin -induced degradation of CXCR4. MDA-MB-231 cells were pretreated with lactacystin for 1 h before being exposed to plumbagin. As shown in Figure 4A, lactacystin had no effect on plumbagin-induced degradation of CXCR4, suggesting that this is an unlikely basis for the suppression of CXCR4 expression by plumbagin.

We also examined the ability of chloroquine, a lysosomal inhibitor, to block plumbagin-induced degradation of CXCR4, as CXCR4 has been shown to undergo ligand-dependent lysosomal degradation [43]. The cells were pretreated with chloroquine for 1 h before exposure to plumbagin. Our results showed that chloroquine at 200 μM only slightly prevented the degradation of CXCR4 (Figure 4B), suggesting that this was arguably not the primary pathway for suppression of expression of CXCR4.

### Downregulation of CXCR4 by plumbagin occurs at the transcriptional level

Since plumbagin did not downregulate CXCR4 expression by enhancing its degradation, we investigated whether suppression occurs at the transcriptional level instead. Cells were treated with plumbagin for different times and then the mRNA was extracted for analysis by real-time PCR. As shown in Figure 4C, plumbagin induced the downregulation of CXCR4 mRNA in a time-dependent manner.

### Plumbagin suppresses constitutive activation of NF-κB in MDA-MB-231 cells

The promoter of CXCR4 is known to contain several NF-κB binding sites [44]. In addition, HER2 oncogene has been shown to activate NF-κB in breast cancer cells [45]. Thus it is possible that plumbagin manifests its effect on CXCR4 by suppressing NF-κB activation. We used a DNA-binding assay to assess the effect of plumbagin on constitutive NF-κB activation in MDA-MB-231 cells, and found that the treatment with plumbagin can suppress constitutive NF-κB activation in a dose-dependent manner (Figure 4D). This result suggests that plumbagin may downregulate CXCR4 expression through inhibition of NF-κB activation. However, DNA binding alone is not always associated with NF-κB-dependent gene transcription [46], suggesting that additional regulatory steps are involved. Subsequent results also indicated that plumbagin inhibited NF-κB reporter activity in a dose-dependent manner in MDA-MB-231 cells (Figure 4E). This hypothesis was also tested simultaneously through the virtual system and confirms that inhibition of NF-κB plays a pivotal role in plumbagin-mediated reduction of CXCR4 (Figure 1C) and other metastatic markers (Figure 1D).

### Plumbagin inhibits binding of NF-κB to the CXCR4 promoter

Whether the downregulation of CXCR4 by plumbagin in MDA-MB-231 cells was due to suppression of NF-κB activation *in vivo *was examined by a ChIP assay targeting NF-κB binding in the CXCR4 promoter. We found that plumbagin suppressed the NF-κB binding to the CXCR4 promoter (Figure 4F), thereby indicating that plumbagin inhibits CXCR4 expression by suppressing NF-κB binding to the CXCR4 promoter.

### Plumbagin suppresses CXCL12-induced breast cancer cell migration and invasion

**Figure 5:**
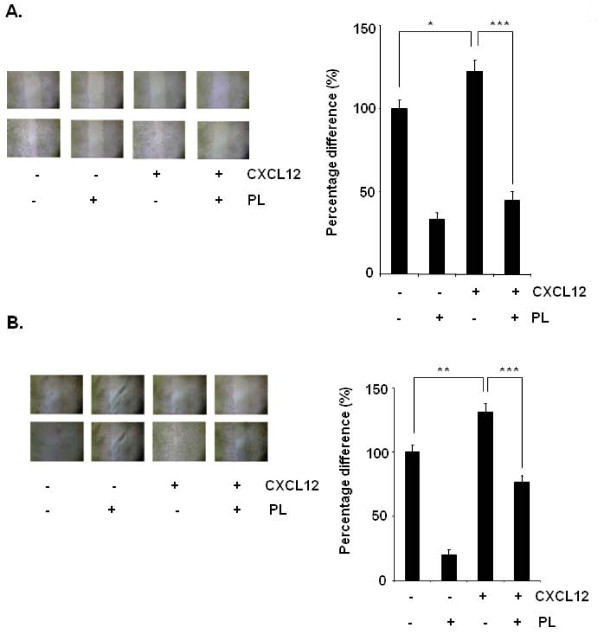
**Plumbagin suppresses migration of breast and gastric cancer cells**. ***A***, The wound-healing assay for evaluating the inhibitory effect of plumbagin on MDA-MB-231 cell migration. Confluent monolayers of MDA-MB-231 cells were scarred, and repair was monitored microscopically after 6 h of pre-treatment with 5 μM plumbagin before being exposed to 100 ng/mL CXCL12 for 24 h. Width of wound was measured at time zero and 24 h of incubation with and without plumbagin in the absence or presence of CXCL12 in RPMI medium containing 1% serum. The representative photographs showed the same area at time zero and after 48 h of incubation. *Graphs*, mean (*n *= 3); *bars*, SE. *, *P *< 0.05. ***B***, The wound-healing assay for evaluating the inhibitory effect of plumbagin on AGS cell migration. Confluent monolayers of AGS cells were scarred, and repair was monitored microscopically after 6 h of pre-treatment with 5 μM plumbagin before being exposed to 100 ng/mL CXCL12 for 24 h. Width of wound was measured at time zero and 24 h of incubation with and without plumbagin in the absence or presence of CXCL12 in RPMI medium containing 1% serum. The representative photographs of three independent experiments showed the same area at time zero and after 48 h of incubation. *Graphs*, mean (*n *= 3); *bars*, SE. *, *P *< 0.05.

**Figure 6:**
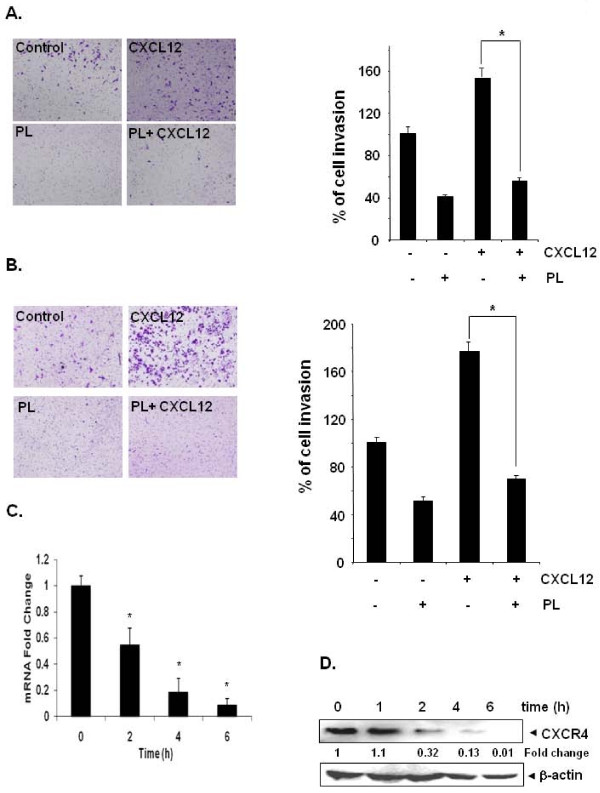
**Plumbagin suppresses invasion in breast and gastric cancer cells**. ***A***, MDA-MB-231 (2 × 10^5 ^cells) were seeded in the top-chamber of the Matrigel. After pre-incubation with or without plumbagin (5 μM) for 6 h, transwell chambers were then placed into the wells of a 24-well plate, in which we had added either the basal medium only or basal medium containing 100 ng/mL CXCL12 for 24 h. After incubation, they were assessed for cell invasion as described in Materials and Methods. Columns indicate mean percentage of invaded cells; bars, S.E. *, *P *< 0.05. ***B***, AGS (2 × 10^5 ^cells) were seeded in the top-chamber of the Matrigel. After pre-incubation with or without plumbagin (5 μM) for 6 h, transwell chambers were then placed into the wells of a 24-well plate, in which we had added either the basal medium only or basal medium containing 100 ng/mL CXCL12 for 24 h. After incubation, the chambers were assessed for cell invasion as described in Materials and Methods. Columns indicate mean percentage of invaded cells; bars, S.E. *, *P *< 0.05. Representative results of three independent experiments are shown. ***C***, Plumbagin suppresses expression of CXCR4 mRNA expression in gastric cancer cells. AGS cells were treated with 5 μM plumbagin for indicated times. Total RNA was isolated and analyzed by RT-PCR assay as described in Materials and Methods. 18S was shown to equal loading of total RNA. Representative results of three independent experiments are shown. ***D***, Plumbagin suppresses expression of CXCR4 protein expression in gastric cancer cells. Cells were incubated with 5 μM plumbagin for indicated times. Whole-cell extracts were prepared and analyzed by Western blot analysis using antibodies against CXCR4. The same blots were stripped and reprobed with β-actin antibody to show equal protein loading. Representative results of three independent experiments are shown.

Whether downregulation of CXCR4 by plumbagin correlates with breast cancer cell migration was examined using an *in vitro *wound healing assay. We found that breast cancer cells migrated faster under the influence of CXCL12 and this effect was abolished on treatment with plumbagin (Figure 5A). Using an *in vitro *invasion assay, we also found that CXCL12 significantly induced the invasion of breast cancer MDA-MB-231 cells and that plumbagin significantly abrogated the invasive activity (Figure 6A).

### Plumbagin inhibits CXCL12-induced gastric cancer cell migration and invasion

In addition, the CXCL12/CXCR4 signaling has been shown to play a critical role in gastric cancer metastasis [12]. Using an *in vitro *wound healing assay, we found that gastric cancer AGS cells migrated faster under the influence of CXCL12 and this effect was abolished on treatment with plumbagin (Figure 5B). To elucidate further the effect on plumbagin on CXCL12-induced cell invasion, we also found that treatment of plumbagin suppressed CXCL12-induced invasion of AGS cells (Figure 6B). We also found that plumbagin also downregulated the expression of both mRNA (Figure 6C) and protein (Figure 6D) for CXCR4 in a time-dependent manner in AGS cells.

## Conclusions

The aim of the present study was to determine whether the anti-cancer agent, plumbagin, can suppress the expression and function of CXCR4, a chemokine receptor that has been closely linked with tumor cell proliferation, invasion, and metastasis. Our results indicate for the first time that plumbagin downregulated the expression of CXCR4 in different types of tumor cells, irrespective of the cell type and HER2 status. For example, plumbagin was found to suppress CXCR4 expression in HER2 overexpressing BT474 breast cancer cells. Our results showed that downregulation of CXCR4 did not occur through proteolytic degradation of the receptor but rather through downregulation of the transcript. Furthermore, suppression of receptor expression led to downregulation of migration and invasion induced by the ligand CXCL12 in both breast and gastric cancer cells.

The CXCR4 chemokine receptor has been found to be overexpressed in different tumors, including breast cancer, gastric cancer, ovarian cancer, glioma, pancreatic cancer, prostate cancer, acute myeloid leukemia, chronic lymphoblastic leukemia (CLL), B-CLL, melanoma, cervical cancer, colon carcinoma, rhabdomyosarcoma, astrocytoma, small-cell lung carcinoma, renal cancer, and non-Hodgkin's lymphoma, as compared to normal cells which show little or no CXCR4 expression [13,22,40,47-49]. Although it is still unclear what leads to the overexpression of CXCR4 in tumor cells, studies point to genetic and microenvironmental factors [50]. PAX3- and PAX7-FKHR gene fusion [51], mutations in the von Hippel Lindau tumor suppressor gene [52], hypoxia in the tumor microenvironment [53], NF-κB [44], and inflammatory cytokines such as vascular endothelial growth factor [54] and tumor necrosis factor alpha [50], have all been implicated in CXCR4 overexpression. Recently, the epidermal growth factor receptor, c-erbB2, and its encoding gene, HER2/*neu*, have also been implicated in the positive regulation of CXCR4 expression at the post-transcriptional level [55,56]. Given that CXCR4 has been linked with the metastasis of various cancers and CXCR4 expression has been correlated with poor prognosis and poor overall patient survival [57], CXCR4 appears an ideal therapeutic target for the investigation of novel therapeutic interventions for the prevention of metastatic cancer.

Our results clearly indicate that plumbagin suppressed CXCR4 expression in both HER2-lacking and -overexpressing breast cancer cells, but had minimal effect on HER2 expression in BT474 breast cancer cells. Our data also showed that plumbagin suppressed CXCR4 expression on various tumor cell lines including gastric cancer, lung adenocarcinoma, renal cell carcinoma, oral and hepatocellular carcinoma, thereby indicating that the effect of plumbagin on CXCR4 is not limited to a single tumor cell type. The ligand-dependent downregulation of the CXCR4 receptor by lysosomal degradation is well documented [40]. Recent reports suggest that degradation involves atrophin-interacting protein (AIP)-4 mediated ubiquitination and degradation [43]. However, our data indicate that plumbagin does not downregulate the CXCR4 through this mechanism. As such, with downregulation of CXCR4 by plumbagin arguably not occurring at the post-translational level, we postulated that the inhibition of CXCR4 expression by this quinine could occur at the transcriptional level. Indeed, we found that plumbagin downregulated the expression of CXCR4 mRNA in breast and gastric cancer cells.

Plumbagin has been previously reported to downregulate NF-κB activation in various tumor cells [23]. Interestingly, the NF-κB binding site has also been identified in the proximal region of the CXCR4 promoter and postulated to play a role in CXCR4 expression in human breast cancer cells [44]. Therefore, it is possible that downregulation of CXCR4 by plumbagin occurs via the suppression of NF-κB. Indeed, we found that inhibition of constitutive NF-κB activation by plumbagin leads to downregulation of CXCR4 in MDA-MB-231 cells. The NF-κB mediated regulation of CXCR4 in the tumor cells was also tested in the virtual system and the predictions corroborated with the experimental results. Whether mechanism(s) other than suppression of NF-κB activation are involved in downregulation of CXCR4 by plumbagin, cannot currently be confirmed or ruled out. Furthermore, besides CXCR4, the activation of NF-κB also induces the expression of various molecules including cyclooxygenase-2, matrix metallopeptidase-9, and adhesion molecules such as intracellular adhesion molecule 1, vascular cell adhesion molecule 1, and endothelial-leukocyte adhesion molecule 1, all of which have been linked with cancer cell migration, invasion, and metastasis [58]. Because plumbagin can inhibit both DNA binding ability and transcriptional activation of NF-κB, as shown in this study, it is possible that plumbagin can suppress the expression of other NF-κB regulated molecules as well in breast cancer cells. We further investigated the effect of plumbagin on CXCL12-induced migration and invasion of both breast and gastric cancer cells. We found that preincubation of cells with plumbagin completely blocked CXCL12-induced migration and invasion of both breast and gastric cancer cells.

Plumbagin has been shown to inhibit different aspects of tumor initiation and progression, including proliferation, invasion, angiogenesis, and metastasis in various tumor cell lines and animal models [23-30]. Our data shows for the first time that plumbagin downregulates expression of CXCR4 in a variety of tumor cells, a key receptor involved in the cross-talk between tumor cells and its microenvironment, and thus, that some of the anti-tumor effects of plumbagin are possibly mediated through CXCR4 regulation. Further *in vivo *studies are being planned to demonstrate the relevance of these observations to cancer treatment.

## List of Abbreviations

CXCR4: chemokine receptor-4; ChIP: Chromatin immunoprecipitation; FBS: fetal bovine serum.

## Competing interests

The authors declare that they have no competing interests.

## Authors' contributions

KAM, MKS, PR, FL, LR, HSH, RK, SNS, SK, BR, PB performed all the experiments. SV, ESK, LHKL, KSA, APK, and GS analyzed the data and wrote the manuscript. All the authors have read and approved the manuscript.
